# External validation of nomogram for predicting malignant intraductal papillary mucinous neoplasm (IPMN): from the theory to the clinical practice using the decision curve analysis model

**DOI:** 10.1007/s13304-021-00999-4

**Published:** 2021-02-23

**Authors:** Riccardo Casadei, Claudio Ricci, Carlo Ingaldi, Alessandro Cornacchia, Marina Migliori, Mariacristina Di Marco, Nico Pagano, Carla Serra, Laura Alberici, Francesco Minni

**Affiliations:** Department of Internal Medicine and Surgery (DIMEC), Alma Mater Studiorum, University of Bologna, S. Orsola-Malpighi Hospital, Via Massarenti n.9, 40138 Bologna, Italy

**Keywords:** Pancreas, Intraductal papillary mucinous neoplasms, Nomogram, Dysplasia

## Abstract

The management of IPMNs is a challenging and controversial issue because the risk of malignancy is difficult to predict. The present study aimed to assess the clinical usefulness of two preoperative nomograms for predicting malignancy of IPMNs allowing their proper management. Retrospective study of patients affected by IPMNs. Two nomograms, regarding main (MD) and branch duct (BD) IPMN, respectively, were evaluated. Only patients who underwent pancreatic resection were collected to test the nomograms because a pathological diagnosis was available. The analysis included: 1-logistic regression analysis to calibrate the nomograms; 2-decision curve analysis (DCA) to test the nomograms concerning their clinical usefulness. 98 patients underwent pancreatic resection. The logistic regression showed that, increasing the score of both the MD-IPMN and BD-IPMN nomograms, significantly increases the probability of IPMN high grade or invasive carcinoma (*P* = 0.029 and *P* = 0.033, respectively). DCA of MD-IPMN nomogram showed that there were no net benefits with respect to surgical resection in all cases. DCA of BD-IPMN nomogram, showed a net benefit only for threshold probability between 40 and 60%. For these values, useless pancreatic resection should be avoided in 14.8%. The two nomograms allowed a reliable assessment of the malignancy rate. Their clinical usefulness is limited to BD-IPMN with threshold probability of malignancy of 40–60%, in which the patients can be selected better than the “treat all” strategy.

## Introduction

Intraductal papillary mucinous neoplasms (IPMNs) may exhibit a spectrum of neoplastic transformation ranging from low-grade dysplasia to high-grade dysplasia (HGD) until invasive carcinomas. Thus, IPMNs have a potential for malignancy, following the “adenoma-carcinoma” sequence, particularly the main duct and mixed forms (50–75%), and to a lesser extent, the BD forms (10–15%) [1–4]. The management of IPMNs is a challenging and controversial issue. The major effort of the physicians was to perform pancreatic resection mainly for malignant IPMNs because pancreatic surgery inherent morbidity and mortality are not negligible [1, 5]. Several guidelines and consensus conferences [6–9] stated the indication of surgery, but the percentage of patients who underwent useless pancreatic resection for non-malignant IPMN remains considerable [10, 11]. Therefore, decision-making treatment is often uncertain. Many authors proposed methodologies based on statistics and probability to identify the patients who need surgical resection properly. Among these, preoperative nomograms, basing on variables significantly related to malignant IPMNs, were built [12–16]. The present study aimed to validate the clinical usefulness of preoperative nomograms reported by Attiyeh et al. [12]. The methodology used was a statistical and probabilistic tool called “decision curve analysis (DCA).”

## Materials and methods

### Study design, patient selection, and nomogram

**Fig. 1:**
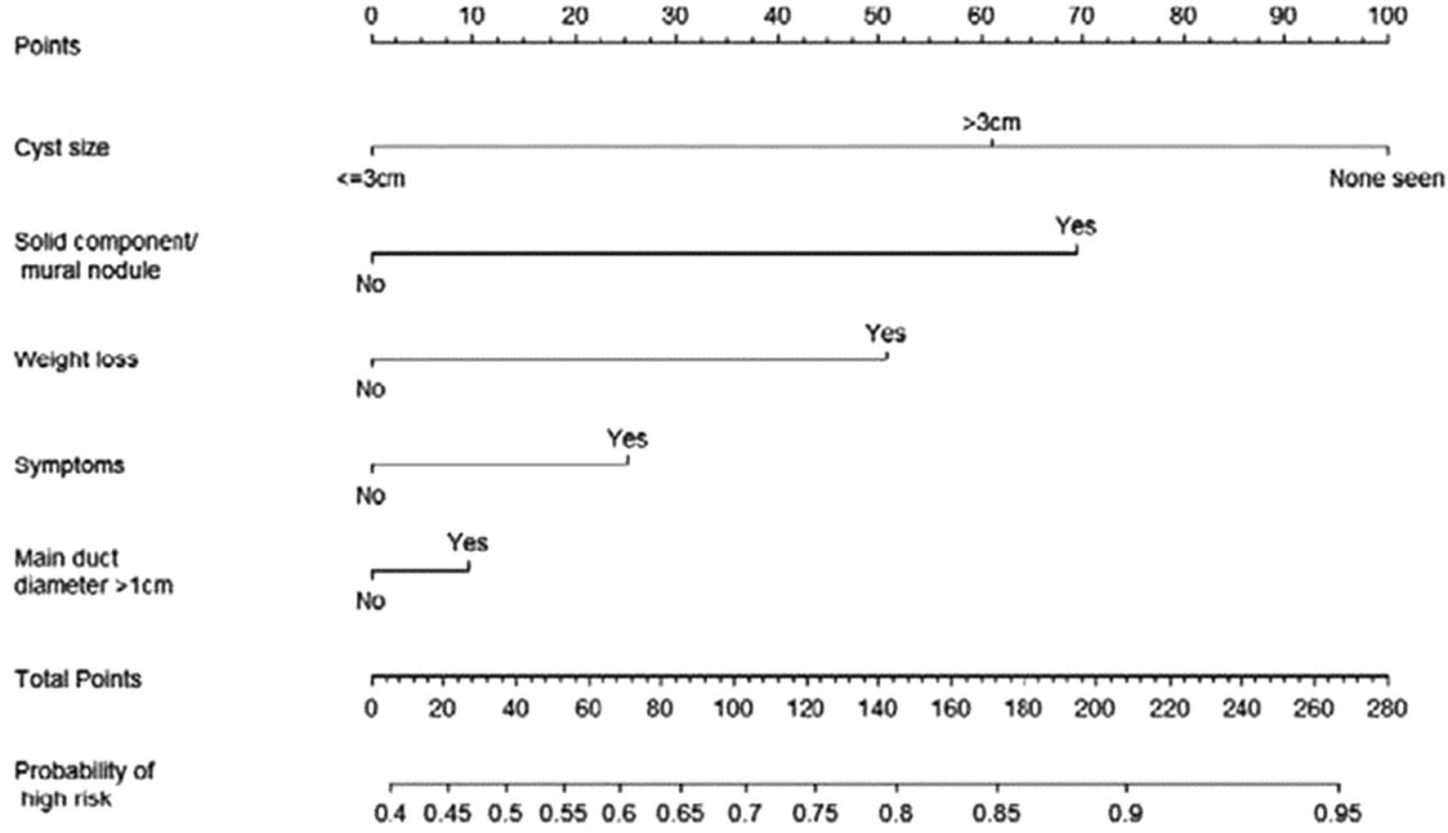
Clinical nomogram for predicting malignancy in patients with MD-IPMN

**Fig. 2:**
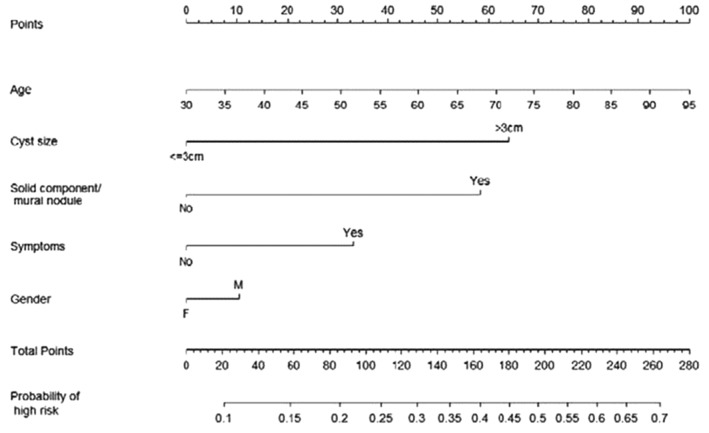
Clinical nomogram for predicting malignancy in patients with BD-IPMN

This is a retrospective study based on a prospectively maintained database of 457 Intraductal Papillary Mucinous Neoplasms (IPMNs) observed from January 2004 to January 2020. The study was approved by the Ethical Committee of S. Orsola-Malpighi Hospital (64/2017/U/Oss) with patient informed consent. The IPMNs types I–II–III were defined according to the consensus conference of Fukuoka 2012 [17]. The diagnostic work-up included Ca 19-9 serum value, Magnetic Resonance Cholangio-Pancreatography (MRCP), and, in selected cases, a multidetector computed tomography (MDCT) and endoscopic ultrasonography (EUS) with or without fine needle biopsy (FNB), were performed. Pancreatic resection was always performed in patients affected by IPMNs with high-risk stigmata according to the consensus conference of Fukuoka 2016 [6] and in selected young patients (< 65 years) with worrisome features. All the other patients underwent surveillance. Only patients who underwent pancreatic resection, both up-front and after a period of follow-up, were included in the analysis because a pathological diagnosis was available. Two nomograms, regarding main and branch duct IPMN, respectively, were evaluated [12] (Figs. 1, 2). For each patient, the data included in the two nomograms were collected: gender, age, symptoms (jaundice and weight loss), tumor site, radiological diagnosis (IPMN types I-III versus type II), solid component/mural enhancing nodules, Wirsung duct size, tumor size, and definitive pathological diagnosis. Also, the type of pancreatic resection and the postoperative data (mortality and morbidity, pancreatic fistula) were reported but not included in the analysis.

### Terminology and definition

Postoperative mortality was defined as the number of deaths occurring during hospitalization or within 90 days after surgery. Postoperative morbidity included all complications following surgery up to the day of discharge, according to the Clavien–Dindo classification [18]. A postoperative pancreatic fistula (POPF) was defined according to the 2016 definition proposed by the International Study Group of Pancreatic Fistula (ISGPF) [19].

### Statistical analysis and description of decision curve analysis

All the categorical variables were described as frequencies and percentages, while the continuous variables were reported as medians and interquartile ranges. The analysis was performed in two steps. First, a calibration of the score was obtained for both the nomograms, calculating the ability of the score in predicting the probability of malignancy of an IPMN. For this purpose, a logistic regression between the score and malignancy rate was carried out. In relation to the small sample, with the aim to reduce the dispersion risk of the curve, an evaluation of the standard error through the technique of “sandwich estimator of variance,” was performed. Moreover, the nomogram score was simplified in the interval of 20 points. The results were reported for each score point as the post-estimation probability of malignancy within a 95% confidence interval (95% CI). A two-sided *P* value < 0.05 indicates, for each point, a significant increase in the probability concerning the previous value. Second, both nomograms were tested concerning their clinical usefulness using the decision curve analysis (DCA) [20–23]. Briefly, decision curve analysis (DCA) is a simple statistical method that allows calculating a clinical benefit for one or more predictions models in comparison to default strategies of treating all or no patients. The DCA includes on the y-axis the “net benefit” and on the *x*-axis the “threshold probability” (*P*_t_). The net benefit of the model is that it correctly identifies which patients performed a pancreatic resection for IPMN high-grade or invasive carcinoma. Threshold probability refers to how the doctor values the threshold probability of IPMN high-grade or invasive carcinoma for each patient that justifies performing a pancreatic resection. We can assume that the threshold probability (*P*_t_) of a disease at which a patient would opt for treatment is informative of how the patient weighs the relative harms of a false-positive and a false-negative prediction. Thus, the net benefit was calculated as follows:

$$\mathrm{Net}={\left(\frac{\mathrm{TP}}{n}-\frac{\mathrm{FP}}{n}\right)\times \left(\frac{{P}_{\mathrm{t}}}{1-{P}_{\mathrm{t}}}\right)}.$$

In this formula, TP and the FP are the numbers of patients with true- and false-positive results, *n* is the total number of patients, and *P*_t_ is the threshold probability of the disease. This theoretical relationship is then used to derive the net benefit of the model across different threshold probabilities. Plotting net benefit against threshold probability yields the “decision curve.” It was tested for three competing strategies: (1) “to treat all” patients with a pancreatic resection, (2) to “treat none” (3) to select the patients for the pancreatic resection using a nomogram. We also tested if some single factor included in the nomograms predominate over the others. The best model will have the highest Net benefit. We also calculated the useless pancreatic resection avoided for each strategy.

## Results

**Fig. 3:**
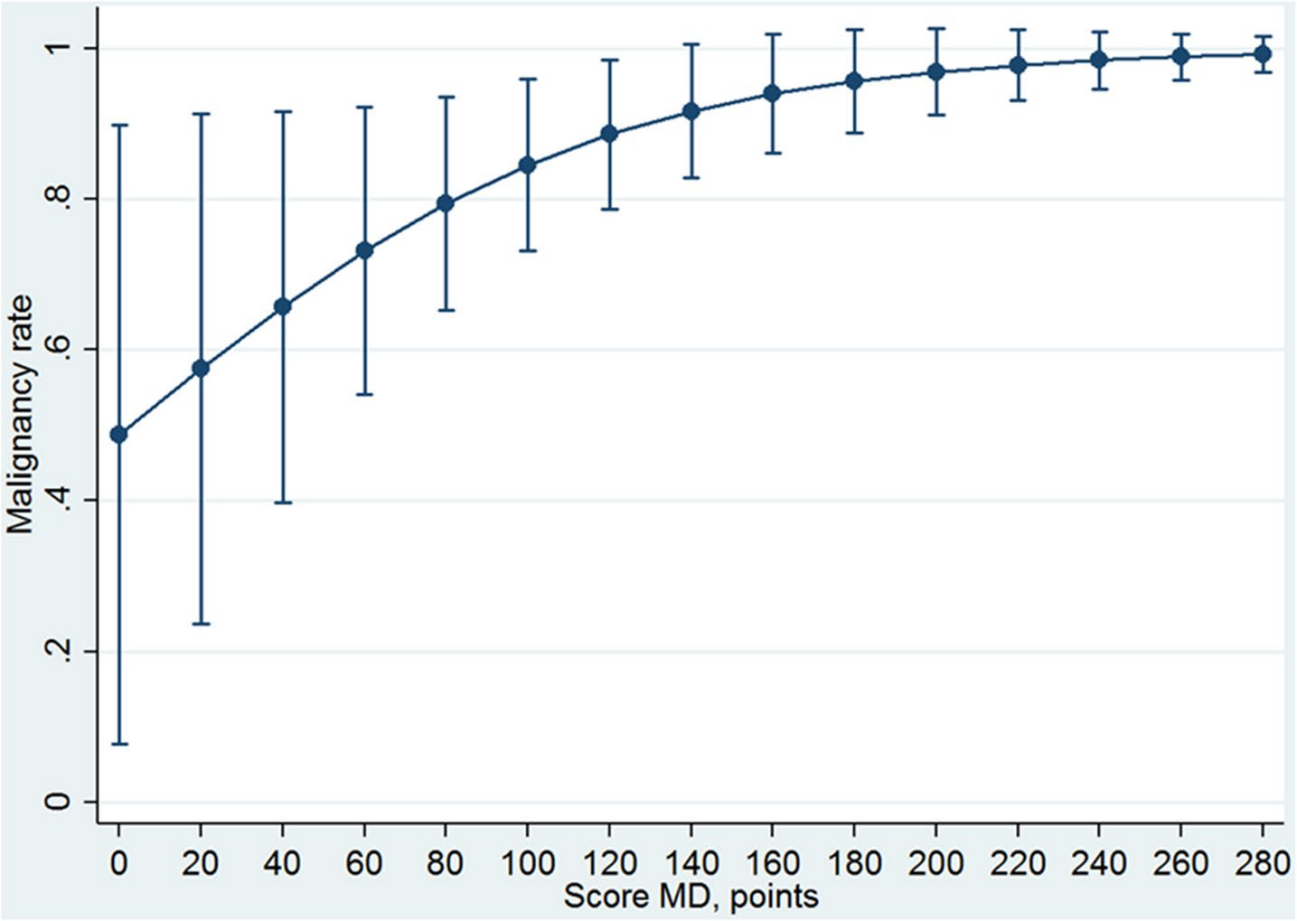
Calibration curve of MD-IPMN nomogram

**Fig. 4:**
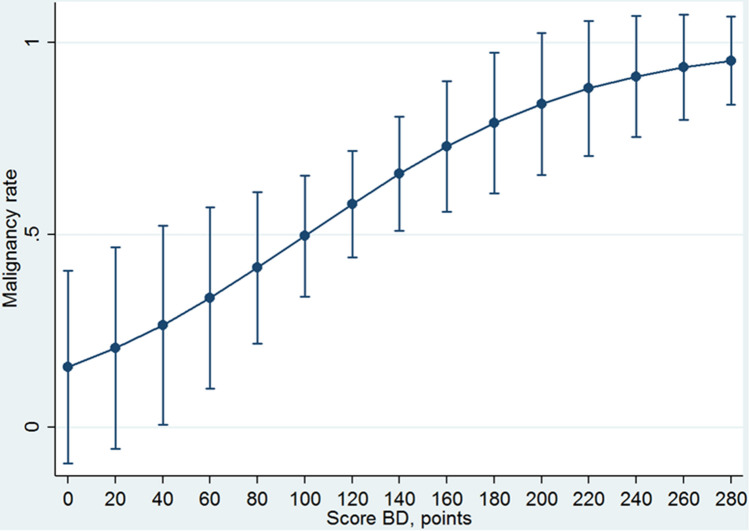
Calibration curve of BD-IPMN nomogram

Four-hundred-and-fifty-seven patients affected by IPMN were observed from January 2004 to January 2020. Of these, 98 patients underwent pancreatic resection with pathological diagnosis and were analyzed. The remaining 357 patients were surveilled and were not analyzed. The characteristics of the patients, type of pancreatic resection, and postoperative results are reported in Tables 1 and 2, respectively. The patients were usually female (52.1%), with a median age of 69.7 years (63.6–74.9). Symptoms were not frequent (38.8%), while jaundice and weight loss were sporadic (8.2 and 9.2%, respectively). IPMN was type II in 57.1% of cases, mainly located in the pancreatic head (32.6%), or diffused to the whole pancreas (39.8%). Mural enhancing nodules were present in 57.1%, and the median main duct size was 5 mm, cyst size was ≤ 30 mm in 61.2%. Pathological diagnosis was mainly IPMN high grade and invasive carcinoma (69.4%): malignancy of MD-IPMN resulted in 79.2% of cases, BD-IPMN in 58.5%. The most frequent pancreatic resection performed was distal pancreatectomy (40.8%), severe complications were detected in 14.3%, postoperative mortality in 4.1%. The incidence of clinically relevant pancreatic fistula (grade B and C) was 15.3%. The logistic regression showed that increasing the score of the MD-IPMN nomogram significantly increases the probability of IPMN high grade or invasive carcinoma (beta coefficient = 0.0017 ± 0.008; *P* = 0.029). The calibration of the MD-IPMN nomogram was reported in Table 3 and plotted in Fig. 3. Each interval of 20 points was significantly related to an increased probability of IPMN high grade or invasive carcinoma. The malignancy rate predicted probability ranges from 48.7% (score = 0 points) to 99.2% (score = 260–280 points). Even if each interval of 20 points is statistically related to the probability of IPMN high grade or invasive carcinoma, starting from score > 140 points, the probability of IPMN high grade or invasive carcinoma increased minimally from 94% (score = 140–159 points) to 99% (score = 260–280 points). The calibration of BD-IPMN nomogram was reported in Table 4 and plotted in Fig. 4. The probability of IPMN high grade or invasive carcinoma was significantly related with the increase of the score (beta coefficient = 0.0016 ± 0.008; *P* = 0.033). The malignancy rate predicted probability ranges from 26% (score = 20–39 points) to 95% (score = 260–280 points). The major increase in the malignancy rate was obtained from 60 to 120 points (from 41 to 66%).

**Table 1:** Baseline characteristics of 98 patients affected by IPMNs included in the analysis

Baseline characteristics	Total patients *n* = 98 (%) or median IQR
Sex	
M	47 (47.9)
F	51 (52.1)
Age (years)	69.7 (63.6–74.9)
Symptoms	
No	60 (61.2)
Yes	38 (38.8)
Jaundice	
No	90 (91.8)
Yes	8 (8.2)
Weight loss	
No	89 (90.8)
Yes	9 (9.2)
Tumour site	
Head	32 (32.6)
Body	13 (13.3)
Tail	14 (14.3)
Diffuse	39 (39.8)
Radiological diagnosis	
Type I–III	42 (42.9)
Type II	56 (57.1)
Mural nodule	
No	42 (42.9)
Yes	56 (57.1)
Main duct size (mm)	5 (3–8)
Cyst size (mm)	
≤ 30	60(61.2)
> 30	38 (38.8)
Histological diagnosis	
Low-medium dysplasia In situ carcinoma Invasive carcinoma	30 (30.6) 36 (35.7) 33 (33.7)

*IPMN* intraductal papillary mucinous neoplasm, *M* male, *F* female

**Table 2:** Post-operative results of 98 operated patients affected by IPMN

Post-operative results	Total patients *n* = 98 (%)
Type of pancreatic surgery	
PD	29 (29.6)
DP	40 (40.8)
TP	26 (26.5)
Atypical resection	3 (3.1)
Complications (Clavien–Dindo)	21 (21.4)
No complications	20 (20.4)
1	40 (40.8)
2	9 (9.2)
3	5 (5.1)
4	3 (3.1)
5 (“in-hospital” mortality)	
90 days mortality	
No	94 (94.9)
Yes	4 (4.1)
POPF rate	
No fistula	75 (76.5)
BL	8 (8.2)
B	12 (12.2)
C	3 (3.1)

*IPMN* intraductal papillary mucinous neoplasms, *PD* pancreatoduodenectomy, *DP* distal pancreatectomy, *TP* total pancreatectomy, *POPF* post operative pancreatic fistula according to 2016 updated ISPGS classification, *BL* biliary leak

**Table 3:** Calibration of MD-IPMN nomogram score

Score for MD-IPMN, points	Malignancy rate predicted probability (95% CI)	*P* value
0	0.487 (0.077–0.897)	Referent
1–19	0.574 (0.236–0.912)	0.020
20–39	0.657 (0.397–0.917)	0.001
40–59	0.731 (0.540–0.922)	< 0.001
60–79	0.794 (0.652–0.936)	< 0.001
80–99	0.845 (0.731–0.959)	< 0.001
100–119	0.886 (0.787–0.985)	< 0.001
120–139	0.916 (0.828–1.000)	< 0.001
140–159	0.939 (0.861–1.000)	< 0.001
160–179	0.956 (0.888–1.000)	< 0.001
180–199	0.969 (0.911–1.000)	< 0.001
200–219	0.978 (0.930–1.000)	< 0.001
220–239	0.984 (0.946–1.000)	< 0.001
240–259	0.989 (0.958–1.000)	< 0.001
260–280	0.992 (0.968–1.000)	< 0.001

*MD-IPMN* main duct intraductal papillary mucinous neoplasm

**Table 4:** Calibration of BD-IPMN nomogram score

Score-BD, points	Malignancy rate predicted probability (95 CI)	*P* value
0	0.015 (0–0.406)	Referent
1–19	0.205 (0–0.468)	0.125
20–39	0.265 (0–0.524)	0.044
40–59	0.335 (0.100–0.571)	0.005
60–79	0.414 (0.216–0.611)	< 0.001
80–99	0.496 (0.339–0.654)	< 0.001
100–119	0.579 (0.441–0.718)	< 0.001
120–139	0.658 (0.510–0.806)	< 0.001
140–159	0.729 (0.560–0.898)	< 0.001
160–179	0.790 (0.607–0.973)	< 0.001
180–199	0.840 (0.655–1.000)	< 0.001
200–219	0.880 (0.705–1.000)	< 0.001
220–239	0.911 (0.753–1.000)	< 0.001
240–259	0.935 (0.798–1.000)	< 0.001
260–280	0.952 (0.837–1.000)	< 0.001

*BD-IPMN* branch duct intraductal papillary mucinous neoplasm

**Fig. 5:**
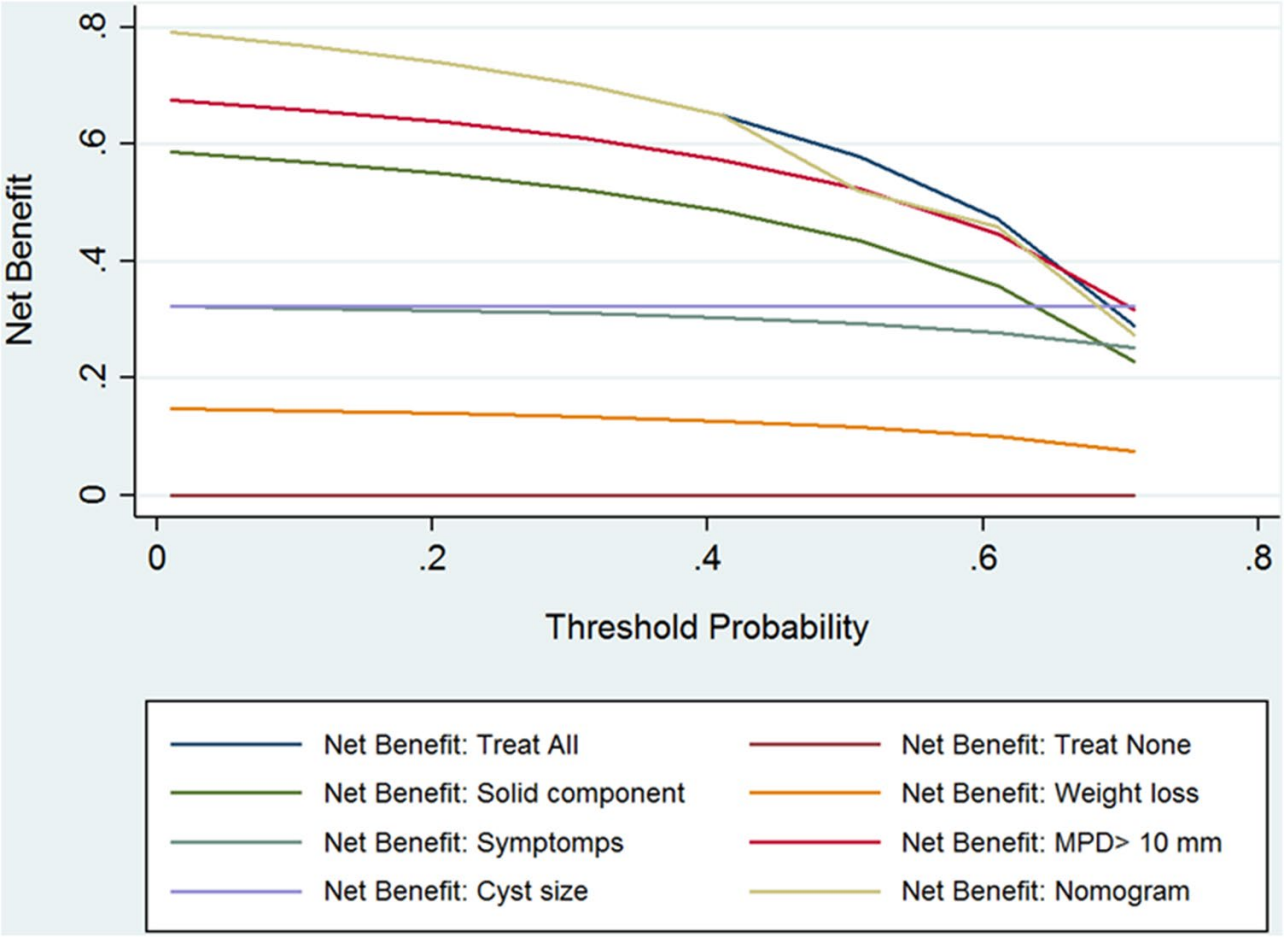
Decision curve analysis of MD-IPMN includes three main strategies: to treat all patients; to treat no patients; to treat the patients using nomogram as instrument of selection. The parameters of nomogram were reported also as single factor. Net benefit represents the patients correctly treated. The threshold probability represents the odd of malignancy for which the physician considered acceptable the surgical risk. The use of nomogram does not provide any advantage for any threshold probability of malignancy

**Fig. 6:**
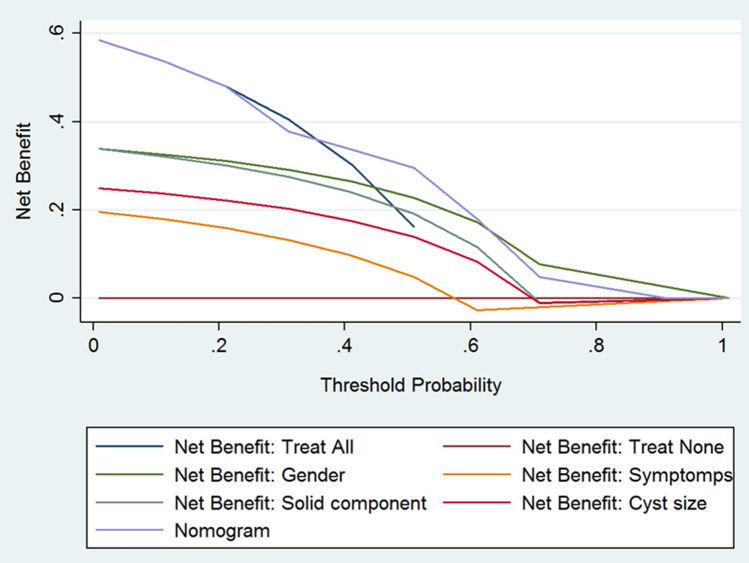
Decision curve analysis of BD-IPMN includes three main strategies: to treat all patients; to treat non patients; to treat the patients using nomogram as instrument of selection. The parameters of nomogram were reported also as single factor. Net benefit represents the patients correctly treated. The threshold probability represents the odd of malignancy for which the physician considered acceptable the surgical risk. The use of nomogram provide some advantage for a range of value 40–60% threshold probability of malignancy

The usefulness of both nomograms was reported in two DCA curves (Figs. 5, 6) for MD-IPMN and BD-IPMN nomograms, respectively. The net benefits and the number of useless pancreatic resection avoided were reported in Tables 5 and 6.

**Table 5:** Net benefit values related to the three approaches in MD-IPMN: “treat all,” “treat none,” and “treat use the nomogram”

Accepted threshold probability of malignancy (%)^a^	Net benefit^b^ “treat all”	Net benefit^b^ “treat none”	Net benefit^b^ “nomogram”	Incremental net benefit nomogram ^c^	Useless pancreatic resection avoided (%)
1	79.2	0	79.2	0	0
10	76.8	0	76.9	0	0
20	73.9	0	73.9	0	0
30	70.1	0	70.1	0	0
40	65.1	0	65.1	0	0
50	57.9	0	57.9	0	0
60	47.2	0	45.9	− 1.3	− 81.99
70	29.1	0	27.3	− 1.6	− 66.2

*MD-IPMN* main ductal intraductal papillary mucinous neoplasm

^a^We did not report values for threshold superior to 70% because the model showed excessive instability and variability for each increment of threshold value

^b^Patients with malignancy correctly treated with surgical resection

^c^The advantage in patients correctly treated with surgical resection using nomogram comparing with treat all strategy

**Table 6:** Net benefit values related to the three approaches in BD-IPMN: “treat all,” “treat none,” and “treat use the nomogram”

Accepted threshold probability of malignancy (%)	Net benefit^a^ “treat all”	Net benefit^a^ “treat none”	Net benefit^a^ “nomogram”	Incremental net benefit nomogram^b^	Useless pancreatic resection avoided (%)
1	58.5	0	58.5	0	0
10	53.8	0	53.8	0	0
20	48.1	0	48.1	0	0
30	40.5	0	37.7	− 2.8	− 6.1
40	30.4	0	33.8	3.3	4.8
50	16.9	0	29.5	13.4	12.9
60	− 5.3^d^	0	17.9	23.2	14.8
70	− 41.6^d^	0	4.7^c^	46.3^c^	18.9^c^
80	− 116.1^d^	0	− 8.1^c^	108^c^	25.3^c^
90	− 356.3^d^	0	0^c^	356^c^	35.2^c^
100	− 400.7^d^	0	0^c^	400.7^c^	40.4^c^

*BD-IPMN* = Main ductal intraductal papillary mucinous neoplasm

^a^Patients with malignancy correctly treated with surgical resection

^b^The advantage in patients with malignancy correctly treated with surgical resection using nomogram comparing with treat all strategy

^c^The strategy based on nomogram tested is not superior with respect to a single parameter (gender) of nomogram itself

^d^The prevalence of the disease was inferior to threshold probability

About MD-IPMN nomogram, Fig. 5 suggested that net values related to the use of nomogram are never superior to those obtained performing the surgical resection in all cases. The net benefit “nomogram” ranged from 79.2 to 27.3%, starting from a threshold probability of 1% until 70%. Net benefit “treat all,” and net benefit “nomogram” resulted similar for the different value of threshold probability of malignancy until the value of 50%. For threshold values of 60% and 70%, the net benefit “treat all” was better than the net benefit “nomogram” (47.2 and 29.1% versus 45.9 and 27.3%, respectively). In addition, useless pancreatic resection avoided resulted 0% and, for value of 60 and 70%, it was negative (− 81.99 and − 66.2%). About BD-IPMN nomogram, Fig. 6 suggested that the use of nomogram produces the highest net benefits only for threshold probability between 40 and 60% (incremental net benefit nomogram = 23.2%). For these values, a maximum of 14.8% of useless pancreatic resection should be avoided. For value inferior to 40%, and superior to 60%, the use of nomogram did not represent the best choice. For threshold value > 70%, the net benefit “nomogram” decreased to 4.7%, − 8.1% and 0%.

## Discussion

Although the 2016 Consensus conference of Fukuoka [6] clearly stated when pancreatic resection is recommended for MD-IPMN and BD-IPMN, the optimal treatment remains controversial. Indeed, a large percentage of patients affected by both MD-IPMN and BD-IPMN who underwent pancreatic resection did not present a malignant IPMN. The effort of this study was to validate the use of two nomograms designed to predict the presence of high-grade dysplasia/invasive carcinoma in both MD-IPMN and BD-IPMN. The DCA method was used because it seems particularly suitable in this setting in which a risk of a wrong choice could be not negligible. The advantage of this model, in contrast, to the standard measures, such as the accuracy, was that the area under the curve (AUC) metric focused solely on the predictive accuracy of a model. In other words, in contrast to AUCs, DCA suggests whether the model is worth using at all or which of other more models is preferable [24]. The present study showed that the two nomograms were statistically well-calibrated because the logistic regressions assessed for both nomograms have a significant ability in predicting the presence of high-grade IPMN or invasive carcinoma, increasing the values of the score. This datum means that the malignancy rate predicted is reliable, and it seems to represent a useful parameter for decision-making treatment. In particular, the first model (related to MD-IPMN) showed that starting from score > 140 points, the probability of IPMN high grade or invasive carcinoma increased minimally. In other words, the prevalence of malignant IPMNs resulted very high for score > 140 points, and further distinction appeared useless. Thus, the nomogram for MD-IPMN is useless from value > 140 points. On the other hand, the second model (related to BD-IPMN) showed a slight increase in the malignancy rate with a delayed plateau (Fig. 4). However, from 60 to 120 points, it seems that the malignancy probability increases strongly (from 41 to 66%). This datum means that, in this interval of points, the patients can be selected in the best way.

The DCA allowed different results regarding the clinical usefulness of the two nomograms. Regarding the MD-IPMN nomogram, it is not able to select furtherly the patients with a high risk of malignancy respect to “treat all” strategy. Also, the nomogram is not useful in avoiding useless pancreatic resection. Finally, for the value of threshold probability > 50%, the nomogram resulted less useful than to “treat all” strategy. Indeed, if we consider suitable for surgery all patients having a risk of malignancy at least (threshold probability) of 70%, the treatment strategy based on nomogram will have a net benefit of 27.3% against the 29.1% for a treatment strategy that provides to treat all the cohort of patients affected by MD-IPMN. In summary, the high rate of malignancy (79.2%) of MD-IPMN makes useless an instrument for the selection of patients, such as the nomogram. Henceforth, the optimal strategy is to perform a pancreatic resection in all the patients affected by MD-IPMN, obviously if fit for surgery, as stated by consensus conference of Fukuoka 2016 [6]. Regarding the nomogram related to BD-IPMN, there are some differences in relation to the threshold probability of malignancy: 1-accepting a low threshold probability of malignancy (< 40%), the nomogram allowed the same results of the “treat all” strategy. In other words, if the main goal is to operate all patients even if the risk of malignancy is low, the nomogram is useless to select the patients adequately; 2-on the contrary, if we considered a threshold probability of malignancy between 40 to 60%, the nomogram allowed a net benefit until 23% respect to the strategy “treat all.” Besides, in this range of value, the nomogram allowed to avoid useless pancreatic resection in 14.8% of cases; 3-finally, if we considered only a very high value of the threshold probability of malignancy (> 60%), the nomogram resulted inferior to the strategy proposed by a single parameter (male gender). Thus, if we decide to operate only patients with a threshold probability of malignancy > 60%, the nomogram is not clinically useful, and it is not able to adequately select the patients for the proper strategy treatment. In summary, the nomogram related to BD-IPMN was clinically useful only in the range between 40 and 60% of the threshold probability of malignancy. Henceforth, a “super-selection” that minimizes close to 0, the useless pancreatic resection and, maximize to 100%, the rate of true positive was not possible with this tool.

The present study has several limitations. First, the models were constructed using a small sample size and retrospective data from a prospective single-center database. Second, this is a surgical population and, thus, for definition already super selected. Nonetheless, the use of the DCA approach and the availability of threshold probability reduced the risk due to selection bias typical of the surgical population.

In conclusion, the two nomograms were statistically well-calibrated, allowing a reliable assessment of the malignancy rate (HGD and invasive carcinoma) of both MD-IPMN and BD-IPMN. However, the nomogram related to the MD-IPMN did not result clinically usefulness because it is not able to make a better selection of patients compared with the treatment strategy “treat all”. On the other hand, the nomogram related to BD-IPMN seems to be clinically useful only in a range of value of the threshold probability of malignancy (40–60%) in which it can select the patients better than the “treat all” strategy.

## Data Availability

The data reported are available and are included in our database of pancreatic neoplasms.

## Code availability

n.64/2017/U/Oss.
